# Mr. Hardwick, on Hydrophobia

**Published:** 1809-01

**Authors:** A. Hardwick

**Affiliations:** Winslow, Bucks


I am, &c.

JOSEPH ARNOLD.

*Nov.* 18, 1808.

---

### *To the Editors of the Medical and Physical Journal.*

GENTLEMEN,

AS your valuable Journal is open for the remarks of those who wish to promote physical knowledge, if the observations of one assuredly inexperienced in the subject of which he is about to treat, are found acceptable, you will much oblige me by inserting them. I wish you to understand that I am perfectly ignorant of the subject, as far as experience goes, and that the following remarks are merely suggested by the careful review of cases that have come under the cognizance of others, and from the candour with which they have been offered to the public, we have no right to question the truth. The medium of your Journal has furnished us with a variety of cases of hydrophobia, sufficient to convince those who have never witnessed the disease, of its real existence. It has furnished us with the whole history of the disease, from beginning to end. It has made us acquainted with the train of symptoms, by which we may infallibly distinguish it from every other disease, and has traced with the greatest accuracy the minutest circumstances attending it. But with all this, the same deficiency exists respecting its cure. Not one step has been advanced towards the point for which these communications were made. We find ourselves now exactly where we began; to our mortification we find that hydrophobia certainly exists, and as certainly proves fatal. As the affair stands at present, the most confident practitioner attacks the disease with diffidence and despair. One unhappy victim follows another, the same train of misery attends them all; and yet clear as this fact is to the senses of the medical man, he perseveres in the *same* unsuccessful plan, nor lets repeated failure rouse him to the adoption of another. The only consolation he can receive from this, is, that others have done so before him. But these are mournful precedents, and I think the urgency of the disease loudly demands their rejection. A wide field is open to the ingenuity of the profession, which cannot be exercised whilst it adheres so firmly to rules, which have nothing for their recommendation but their frequent adoption. The plans your correspondents have adopted, (which differ but little from each other) are highly spirited, and though unsuccessful do honour to their respective Inventors. It is satisfactory to reflect that we have done our best, however mortifying the result; but I cannot conceive any merit attached to that man, who perseveres, in spite of conviction, to adopt measures which at best are useless and probably injurious. Injurious, because the violent action they necessarily induce, tends to exhaust the system, without acting specifically on the disease. Mercury has been resorted to with the most sanguine hopes that humanity could inspire, but alas hydrophobia is unlike lues, and mercury, the undoubted specific in the latter, has afforded ample proof of its inefficacy in the former. Why then continue its use? Has a single symptom been relieved by it? When it has affected the system, (and this must be the ultimate intention of its use) it uniformly increases the discharge from the salivary glands. This I think must aggravate one symptom, and not the least distressing one, the attempts to disengage the mucus from the throat, and the incessant cough which these attempts naturally produce. But I do not allude to mercury alone, small doses of camphor, ammonia, monia, opium, &c. if not so injurious, prove at least equally useless. When I say small doses, I mean to speak comparatively with their effects; for two grains of opium, though a large dose for a man in health, or under common disease, is comparatively very trifling to one under the influence of tetanus or rabies canina. Security in the first instance suggested the cautious use of powerful medicines, and the safe determination of doing no mischief where the advantage was uncertain, prompted very properly caution in their administration. But this will hold good no longer. We hear of two, three, four and five grains of opium being swallowed without any sensible effect. Our intention is to give relief; and if we determine upon the use of this drug, why not administer it in the largest doses consistent with the safety of our patient? Had I ventured to give five grains (and this should certainly be the *smallest* dose I would use in this disease) without perceiving injurious effects from it, would there be any rashness in increasing my next dose to eight grains, and so on to ten or upwards? In your last number a case is related by Mr. Thomson, with whom I have a slight personal acquaintance, and for whose abilities I have the highest esteem, where a large quantity of opium was used, and followed up in a most spirited manner, without producing any sensible effect whatever. The same practice has been adopted by others with the same result. I conceive these precedents sufficient to authorise us in the use of opium on a more enlarged scale, and if we do but give relief, we shall have advanced one step further than experience has hitherto brought us. Your ingenious correspondent, who signs himself C. B. has certainly made a mistake, though it appears wholly unintentional. He says, that "five days" from the commencement of "unambiguous symptoms" of canine hydrophobia, is a term of from "thirty-six to forty-eight" hours longer than any person has been known to survive under them. I take it for granted that this has been uniformly the case in his practice; but the assertion is too general, and no doubt candour will induce him to correct it. In a case which came under the observation of my father a twelvemonth ago, the characteristic symptoms, to wit, horrors and convulsive spasms at the sight of liquids, with total inability of swallowing them, were perfectly evident on Tuesday morning, and the patient survived till the following Saturday, P. M. The same treatment was resorted to in this case, and though followed up with the greatest alacrity, not a symptom was relieved by it. The body was opened a few a few hours after death, and not the least unusual appearance could be found in any part of it, except a degree of fulness in the vessels of the brain, which might probably arise from the violent convulsion in which the patient expired. This melancholy case has afforded me one proof of the inefficacy of caustic, when applied thirty-six hours after the bite.

A boy, 10 years old, was bitten in the lip by a dog, with which he was accustomed to play. This happened in the evening. The boy was brought to me the next night, but from a gross misrepresentation on the part of his parent, no notice was taken of it till the following morning. Caustic was then applied, and repeated for several days. It was at last discontinued, and the parts allowed to cicatrize. Ten weeks from this time the disease appeared, and terminated fatally on the fifth day. I should be happy to relate the case at full, but regular notes were not taken. The case was witnessed by many professional men of the first respectability. I hope it will not be deemed presumptious, if I here object to the practice of frequently endeavouring to make the unfortunate patient swallow liquids. To be sure we sometimes succeed in getting a little down, but is not the attempt attended with worse consequences, than we can possibly derive benefit from it? I cannot help thinking it cruel in the extreme, and fear that the experiment has been made more from motives of curiosity, on the part of the practitioner, than from any expectation of serving the patient. Surely it is our duty to sacrifice every selfish motive, and humanity forbids the repetition of this painful trial. It is a comfortable reflection that antidotes have been found for many animal poisons; this is sufficient to inspire the hope, that accident will some day afford us a remedy for hydrophobia; I say accident, because it is chiefly to accident that we are indebted for antidotes to other poisons. Improved as the science of physiology is, it does not afford us one satisfactory theory on the nature of this disease. We see its phenomena, but cannot trace them to their cause. We endeavour to explain them by the rationale of other diseases, (in which by the bye we are too often foiled) but are lost in the very attempt. One train of thought leads us on to another, till we find ourselves at a greater distance from our object than when we first set out. A dark mysterious veil enwraps the subject, which our present impotent exertions have been unable to remove. On accident therefore I ground this hope, the most fragile footing I can find, but, like the drowning victim, tim, I catch at a straw, I anticipate a hope which present facts will scarcely authorise.

Brown's doctrine of overcoming diseased action by inducing stronger action, appears to have swayed every practitioner who has had opportunities of treating this dreadful malady. The attempt was meritorious, but the result fatal. Suppose the scene is now changed, and instead of stimulating the constitution, whether by mercury or otherwise, we endeavour to reduce that action which is already too violent. It will be said, the pulse is often small and quick, and the asthenic plan may therefore debilitate too much. True, but weak as the pulse may be, the spasm is very strong; the nervous energy is impaired, but the muscular contractile power is increased. In short, I had rather see my patient die of debility, than be carried off by an hydrophobic convulsion. The experiment may be worth the trial, however strong our apprehensions for its success. In spasmodic diseases, I have frequently witnessed the good effects of tobacco. May not this also be worth a trial. By moistening this drug, and inserting it into the rectum, it is not impossible that the spasms may be relieved. But this is all conjecture, and I scarcely feel myself authorised to intrude upon your time. I trust however, the purity of my motive will plead my apology. If the preceding observations should stimulate any of your readers to prepare themselves to meet this formidable disease, and to resolve on their plan of treatment before the opportunity of adopting it may occur, I shall feel gratified, even should the effect of such resolution prove abortive.

I am, &c.

A. HARDWICK.

